# Cytokine production in patients with recurrent acute tonsillitis: analysis of tonsil samples and blood

**DOI:** 10.1038/s41598-020-69981-1

**Published:** 2020-08-03

**Authors:** Katharina Geißler, Cynthia Weigel, Katja Schubert, Ignacio Rubio, Orlando Guntinas-Lichius

**Affiliations:** 1 Department of Otorhinolaryngology, Jena University Hospital, Am Klinikum 1, 07747 Jena, Germany; 2 Integrated Research and Treatment Center, Center for Sepsis Control and Care, Jena University Hospital, Jena, Germany; 3 Leibniz Institute for Natural Product Research and Infection Biology – Hans Knöll Institute (HKI) Jena, Jena, Germany; 4 Department of Anesthesiology and Intensive Care Medicine, Jena University Hospital, Jena, Germany; 5 Present Address: Department of Biochemistry and Molecular Biology, Virginia Commonwealth University School of Medicine, Richmond, USA

**Keywords:** Immunology, Diseases

## Abstract

The aim of this study was to examine T cell function in tonsils of patients with recurrent acute tonsillitis (RAT) or peritonsillar abscess (PTA) by analyzing the cytokine production following T cell receptor (TCR) and co-receptor stimulation with a combination of anti-CD3 and anti-CD28 antibodies. The release of IFN-γ, TNF-α, IL-2, IL-4, IL-6, IL-10 and IL-17A from isolated, stimulated T cells of 27 palatine tonsils (10 RAT, 7 PTA, 10 tonsils without inflammation) was measured via a bead-based flow cytometric analysis. The results were compared with the cytokine release of isolated peripheral T cells in a subset of the same patients (6 PTA, 4 patients without signs of inflammation in the blood). TCR stimulation increased the concentration of released cytokines in tonsil and blood as well as in different forms of inflammation and tissue with no inflammation. Stimulation increased the pro-inflammatory cytokines TNF-α, IFN-γ, and IL-2 more than the anti-inflammatory cytokines IL-4 and IL-10 in tonsil and blood samples in RAT, PTA, and samples without inflammation. Blood of patients with PTA showed a higher pro-inflammatory cytokine level compared to the samples of patients without inflammation. T cells in tonsils are fully responsive and competent for antigen-induced cytokine production in RAT and PTA. One should be aware that tonsillectomy, if indicated, might remove a functioning immune organ. Tonsillotomy might be an alternative even in adults to maintain immunological function.

## Introduction

The palatine tonsils as paired lymphatic organs are located at the beginning of the upper aerodigestive tract and are responsible for immune protection against ingested and inhaled pathogens. In this context, innate and adaptive immune reactions interact, whereby tonsillar T cells located primarily in the extra-follicular spaces, in particular, play a dominant role^1^. Cytokines are produced in the context of inflammatory reactions by immune-competent cells. A distinction is made between genuine pro-inflammatory, e. g. tumor necrosis factor (TNF)-α, interferon (IFN)-γ, interleukin (IL)-2, IL-6, IL17-A, and mostly anti-inflammatory cytokines like IL-4 and IL-10^2–4^. Furthermore, depending on the inflammatory scenario, T-cells can differentiate into differently primed states, including Th1, Th2, and Th17. Briefly, Th1 helper cells produce large amounts of IFN-γ, IL-2, and TNF-β, which trigger macrophages and are important for cell-mediated immunity and phagocyte-dependent immune responses. Th2 cells produce IL-4, IL-5, IL-10, and IL-13, which promote antibody production, eosinophil activation, and inhibition of several macrophage functions^5^. Th17 cells and their effector cytokines IL-17A, IL-17F, IL-21, and IL-22 mediate host defense mechanisms to a variety of infections, especially of bacterial origin, and are furthermore involved in the pathogenesis of many autoimmune diseases^6^.

Recurrent acute tonsillitis (RAT) is a common chronic inflammation of the palatine tonsils, which often needs surgical excision^7^. Criteria for tonsillectomy in Germany are at least 6 episodes of tonsillitis per year with antibiotic treatment^8^. Typical symptoms for patients with RAT are a pain in the throat, fever, *foetor ex ore*, and cervical lymphadenopathy. Complications can be local to systemic and include retropharyngeal or peritonsillar abscesses (PTA), sepsis, endocarditis, and glomerulonephritis. PTA describes a peritonsillar localized, deep infection^9^. The infection may cause progressive disease, airway obstruction, sepsis to death.

RAT and PTA or some therapy-refractory systemic diseases, for example, certain forms of psoriasis or IgA-nephropathy^10,11^, are common indications for tonsillectomies. Indeed, tonsillectomy is one of the most common surgical procedures in young adults and infants (e.g. in 2018 Germany-wide 61.336 tonsillectomies with no adenoidectomy and 12.743 tonsillectomies with adenoidectomy^12^. However, despite its large clinical impact, the importance of the palatine tonsil for the immune system and the consequences of its removal are still unclear and controversially debated^13^.

Recently, while characterizing T cell function from palatine tonsils in patients with chronic tonsillitis, we identified differences in the proportions of T cell subpopulations and T cell function in tonsil tissue between various infection scenarios^13^. In the current work, we placed the focus on a more extensive characterization of the cytokine response of isolated and stimulated tonsillar T cells compared side-by-side to blood samples of patients with RAT, PTA, and no inflammation in tonsil tissue and blood.

## Materials and methods

### Patients and tonsillectomy

The study protocol conformed to the ethical guidelines of the 1975 Declaration of Helsinki. It was approved by the ethical review committee from the medical faculty of the Friedrich-Schiller-University Jena (No. 3972-01/14). Written informed consent was obtained from all patients before enrollment.

All patients underwent bilateral tonsillectomy, of note, also in patients with PTA in which both the side with the abscess and the healthy contralateral side were removed. Patients included in this study underwent immediate surgery after presentation without prior incision or drainage/needle aspiration. They did not receive steroids due to swelling before tonsillectomy. Exclusion criteria for all groups were steroid or other immunosuppressive therapy, severe chronic diseases in medical history, and therapy with anticoagulants or coagulation values lying under standard average values. Tonsillectomy was performed in the department of otorhinolaryngology of the Jena University Hospital, Germany. The patients were recruited between September 2013 and September 2014.

13 male and 7 female adult patients with a median age of 31.6 years were included in this study (see Table 1 for patient characteristics). 10 patients presented with RAT with the last episode of tonsillitis at least 3 weeks ago, 7 with PTA (note that the infected and the corresponding healthy tonsil were processed separately) and 3 with tonsil hyperplasia, together with the healthy sides of PTA used as the control group. The cytokine release of isolated T cells was analyzed in all 27 tonsil tissue samples (10 RAT, 7 PTA, 10 tonsils with no inflammation stemming from the healthy sides from PTA and hyperplasia). The results were compared with the cytokine release of isolated T lymphocytes of the blood drawn at the time point of surgery from a subset of 10 patients: 6 PTA and 4 patients with no sign of inflammation (2 patients with RAT and 2 patients with tonsil hyperplasia with no increased inflammation markers like leucocytes or C-reactive protein). For the sake of clarity, from here on, T-cells from tonsillar or blood origin are tagged by the suffix – T (tonsils) and – B (blood), respectively.

**Table 1:** Patients’ characteristics.

	All	RAT	PTA	NO-INFL	p
Gender					
Male	13	7	4	2	0.194
Female	7	3	3	1	0.275
Age, years (median)	31.6	26.5	33.0	35.3	0.136

*RAT* recurrent acute tonsillitis, *PTA* peritonsillar abscess, *NO INFL* no inflammation.

### T cell purification from tonsils

T cells were isolated following a protocol optimized for tonsillar T cells that has been published before^14^. Briefly, tonsil tissue is minced, homogenized, and pressed through a cell strainer. Mononuclear leucocytes were enriched by density gradient centrifugation on Ficoll Histopaque-1077 (Sigma-Aldrich, Steinheim, Germany) and T cells are isolated out of the resulting mononuclear fraction via automated magnetic sorting on the AutoMACs (Miltenyi Biotec, Bergisch Gladbach, Germany) using combined positive selection on CD4 and CD8 magnetic beads (Miltenyi Biotec, Bergisch Gladbach, Germany). The purity of final T cell preparations ranged 90–95%, as ascertained by surface CD3 staining via flow cytometry^13^. T cells were eluted into the cell culture medium (RPMI 1,640 medium supplemented with penicillin and streptomycin and 10% heat-inactivated fetal calf serum (FCS) and kept in a humidified atmosphere at 37 °C and 5% CO_2_ for 24 h before stimulation. Peripheral T cells from a donor and patient blood were isolated and cultured in the same way out of PBMCs, except that a step of erythrocyte lysis was included before magnetic sorting^15^.

### Antibodies and T cell stimulation

T cell stimulation was performed after isolation via two different protocols, one each for low-grade and high-grade TCR stimulation for 48 h. For low-grade TCR stimulation, T cells were stimulated with soluble anti-CD3 and anti-CD28. To this end, cells were challenged with 1.7 μg each of biotinylated anti-CD3ε (clone OKT3), and anti-human CD28 purified (clone CD28.2), both from eBioscience Inc. (San Diego, USA). High-grade stimulation was accomplished using surface-immobilized anti-CD3/CD28 coated on beads. To this end, T cells were challenged with 10 µl of anti-biotin MAC SiBead Particles loaded with biotinylated anti-CD2, anti-CD3, and anti-CD28 per 10^6^ cells (T Cell Activation/Expansion Kit, Miltenyi Biotec, Bergisch Gladbach, Germany), following the kit’s instructions. As a positive control, T cells were treated with a mix of 0.3 µM phorbol 12-myristate 13-acetate (PMA) and 1 µg/ml ionomycin, which circumvents the T cell receptor and receptor-proximal steps to activate the T cell signaling machinery.

T cells were purified from patient tonsils by an optimized and streamlined protocol^14^ and have been previously characterized with regard to T cell subclasses (CD4/CD8) and activation markers^13^.

### Cytokine measurements

Levels of TNF-α, IFN-y, IL-2, IL-4, IL-6, IL-10 and IL-17A in the supernatants of stimulated T cells were analyzed via a magnetic bead-based array using the BD Cytometric Bead Array (CBA)-Human Th1/Th2/Th17 Kit, following the manufacturer´s instructions. All flow cytometry data were acquired using a FACS Canto or FACS Calibur (BD Pharmingen™, Franklin Lakes, USA). Data processing was done with BD Cytometric Bead Array (CBA) FCAP Array Software.

### Statistical analysis

Results are presented as means ± standard deviation (SD), if not otherwise indicated. For comparison between two groups, a unpaired t-tests and for multiple comparison, a one-way-ANOVA with post-hoc Bonferroni correction were performed by using IBM SPSS statistics software version 23.0.0.0, SPSS Inc., Chicago, IL. The level of significance was set to p < 0.05.

## Results

**Figure 1:**
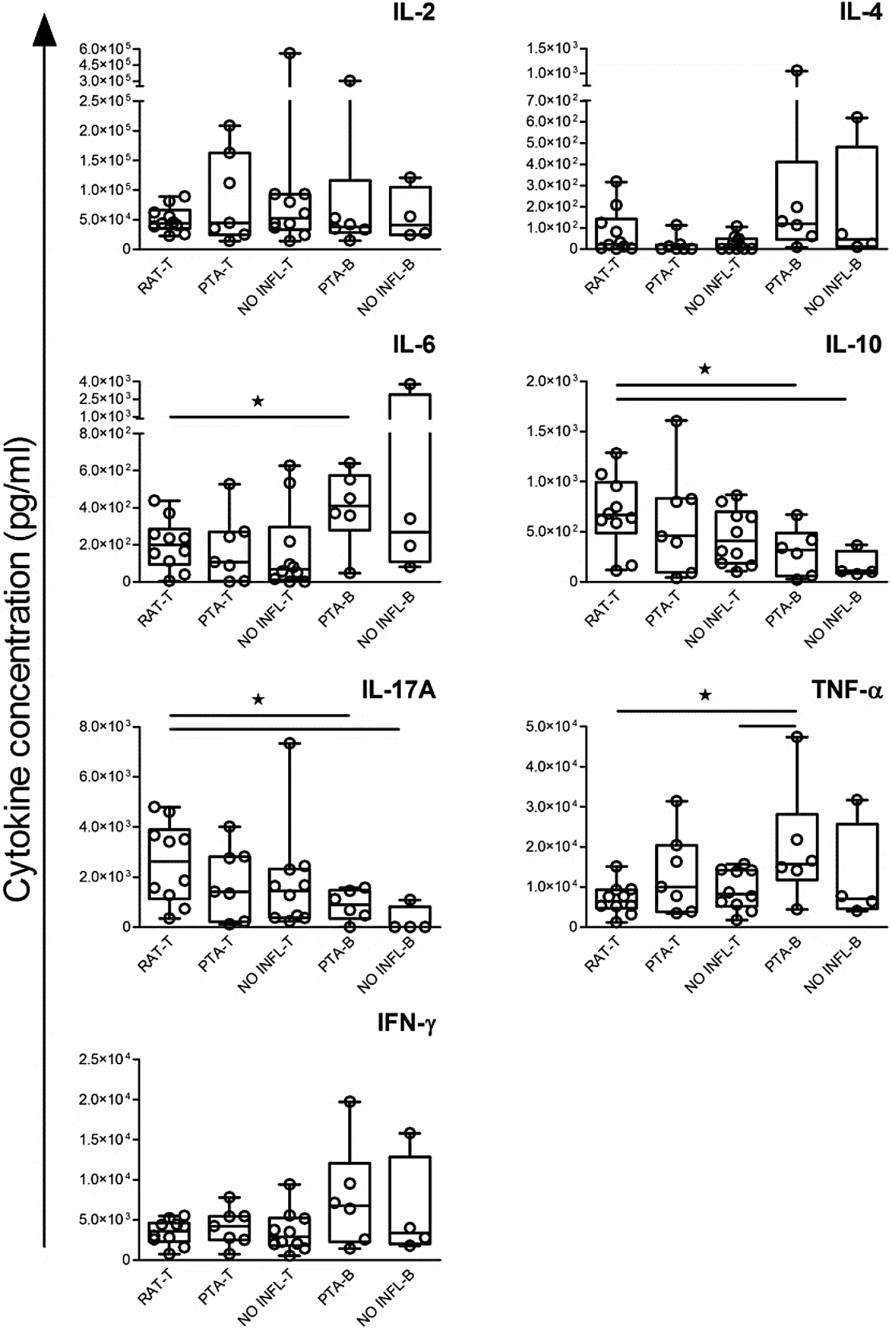
Cytokine release (pg/ml) from purified tonsillar (− T) or peripheral (− B) T cells stimulated with anti-CD3/CD28 Abs immobilized on beads. Data are presented as box plot; circles stand for individual patients, *Significant difference (p < 0.05).

Stimulation of tonsillar T cells with soluble anti-CD3/CD28, bead-immobilized anti-CD3/CD28, and PMA/ionomycin increased the concentration of released cytokines (Table 2 and Supplementary Fig. 1). TCR stimulation led to a stronger increase of genuine pro-inflammatory cytokines TNF-α, IFN-γ, and IL-6 than anti-inflammatory cytokines, prominently IL-4 and IL-10 (Fig. 1, Table 2). Blood of patients with PTA had a higher concentration of pro-inflammatory cytokines in comparison to patients with no sign of inflammation in the blood. Importantly, the secretion of IL-2, the autocrine mitogen responsible for clonal T cell expansion, was more markedly upregulated by immobilized versus soluble anti-CD3/CD28 (Table 2), consistent with many previous studies. Also, T cells from non-inflamed tonsil tissue featured the strongest IL-2 response, in contrast to all other measured cytokines, which were either equally or more prolifically generated by T cells from inflamed tonsils. Tonsil tissue of RAT reacted less after stimulation (Fig. 1).

**Table 2:** Cytokines (pg/ml plotted as mean ± SD) released by tonsillar T cells challenged as indicated.

	RAT-T (n = 10)	PTA-T (n = 7)	NO INFL-T (n = 10)	PTA-B (n = 6)	NO INFL-B (n = 4)	p (Bonferroni correction)
**IL-2**						
Unstimulated	0	0	0	0	0	n.s
CD3/CD28	381.4 ± 345.1	255.1 ± 496.8	421.3 ± 783.6	185.2 ± 261.3	208.9 ± 165.8	n.s
Beads	50,443.7 ± 21,874.2	86,054.0 ± 76,247.0	105,209.5 ± 162,994.7	80,631.3 ± 110,967.2	57,084.3 ± 45,072.6	n.s
PMA/ionomycin	79,069.8 ± 57,901.4	114,886.7 ± 75,916.2	102,692.2 ± 49,564.1	70,979.6 ± 42,542.3	53,411.8 ± 77,095.0	n.s
**IL-4**						
Unstimulated	0	0	0.01 ± 0.04	0	0	n.s
CD3/CD28	2.9 ± 3.2	3.1 ± 5.1	2.9 ± 3.9	36.6 ± 13.2	22.8 ± 33.1	RAT-T vs. PTA-B 0.0001, PTA-T vs. PTA-B 0.0001, NO-INFL-T vs. PTA-B 0.0001
Beads	79.7 ± 107.2	20.7 ± 41.4	25.5 ± 35.6	260.9 ± 395.2	181.5 ± 294.6	n.s
PMA/Ionomycin	44.4 ± 131.4	0	3.0 ± 6.7	20.8 ± 32.5	138.1 ± 276.1	n.s
**IL-6**						
Unstimulated	1.9 ± 3.5	0.07 ± 0.2	0.3 ± 0.6	90.9 ± 171.2	1892.1 ± 2,277.1	n.s
CD3/CD28	29.6 ± 26.0	6.0 ± 10.1	6.7 ± 9.5	321.0 ± 303.3	3,623.4 ± 4,048.3	RAT-T vs. NO INFL-T 0.02, RAT-T vs. PTA-B 0.008, RAT-T vs. NO INFL-B 0.01, PTA-T vs. PTA-B 0.03, NO INFL-T vs. PTA-B 0.007, NO INFL-T vs. NO INFL-B 0.02
Beads	201.6 ± 136.1	177.1 ± 187.1	167.4 ± 227.7	403.0 ± 205.3	1,099.2 ± 1,790.7	RAT-T vs. PTA-B 0.03
PMA/ionomycin	472.7 ± 638.7	207.8 ± 293.6	321.3 ± 299.9	377.6 ± 524.0	193.7387.4	n.s
**IL-10**						
Unstimulated	0.2 ± 0.6	0	0.003 ± 0.009	0.3 ± 0.6	10.1 ± 15.1	n.s
CD3/CD28	27.9 ± 16.9	17.5 ± 19.8	21.8 ± 17.5	37.8 ± 39.7	28.4 ± 27.9	n.s
Beads	685.5 ± 365.2	599.9 ± 539.1	449.7 ± 278.6	298.1 ± 237.6	161.3 ± 135.0	RAT-T vs. PTA-B 0.04, RAT-T vs. NO INFL-B 0.02
PMA/ionomycin	127.1 ± 212.9	2.8 ± 7.5	19.4 ± 31.8	0	32.1 ± 64.2	n.s
**IL-17A**						
Unstimulated	0	0	0	0	0	n.s
CD3/CD28	72.1 ± 54.8	56.7 ± 97.5	50.7 ± 89.0	102.4 ± 104.2	13.3 ± 26.7	n.s
Beads	2,578.2 ± 1611.5	1812.7 ± 1,443.6	1811.2 ± 2,109.5	884.0 ± 609.0	271.8 ± 543.5	RAT-T vs. PTA-B 0.03, RAT-T vs. NO INFL-B 0.02
PMA/ionomycin	1,265.8 ± 2,218.8	0	24.8 ± 78.4	0	715.2 ± 1,430.4	n.s
**TNF-α**						
Unstimulated	0.1 ± 0.3	0.1 ± 0.3	0.3 ± 0.6	0.2 ± 0.4	29.7 ± 32.6	n.s
CD3/CD28	230.9 ± 192.3	262.6 ± 334.8	251.9 ± 260.7	577.6 ± 327.4	706.5 ± 587.7	RAT-T vs. PTA-B 0.02, RAT-T vs. NO INFL-B 0.035
Beads	6,940.0 ± 3,861.2	13,350.5 ± 10,131.2	9,237.3 ± 4,965.6	19,904.0 ± 14,610.4	12,494.6 ± 12,942.6	RAT-T vs. PTA-B 0.02, NO-INFL T vs. PTA-B 0.049
PMA/ionomycin	2,791.5 ± 2,985.3	3,798.3 ± 1929.3	3,805.7 ± 1972.8	9,236.7 ± 5,529.6	6,252.0 ± 10,994.1	RAT-T vs. PTA-B 0.008, PTA-T vs. PTA-B 0.03, NO INFL-T vs. PTA-B 0.01
**IFN-γ**						
Unstimulated	0	0	0	0	0	n.s
CD3/CD28	79.3 ± 60.1	110.6 ± 174.9	68.0 ± 68.8	492.1 ± 682.5	288.4 ± 446.0	n.s
Beads	3,445.2 ± 1558.5	4,139.0 ± 2,344.6	3,567.4 ± 2,615.7	7,801.4 ± 6,568.6	6,084.1 ± 6,545.1	n.s
PMA/Ionomycin	8,831.1 ± 6,328.2	5,981.5 ± 3,026.4	9,340.2 ± 7,815.9	11,065.3 ± 7,421.2	10,577.5 ± 15,586.0	n.s

T cells were purified from patients with recurrent acute tonsillitis (RAT-T), peritonsillar abscess (PTA-T), no inflammation (NO INFL-T), and blood of adult patients with PTA (PTA-B) and no inflammation (NO INFL-B).

*CD* cluster of differentiation, *IFN* interferon, *IL* interleukin, *PMA* Phorbol 12-Myristat 13-Acetat, *SD* standard deviation.

In general, we observed a clear preference of tonsillar versus peripheral T cells for distinct cytokines. Thus, tonsillar T cells of RAT produced higher levels of IL-10 and IL-17 than peripheral T cells (Fig. 1, IL10 RAT-T vs. PTA-B p = 0.04, IL10 RAT-T vs. NO INFL-B p = 0.02, IL17A RAT-T vs. PTA-B p = 0.03, IL17A RAT-T vs. NO INFL-B p = 0.02). At the same time, as expected peripheral T cells were more prolific in the generation of genuine pro-inflammatory cytokines such as TNF-α and IFN-γ after stimulation with soluble anti-CD3/CD28 and immobilized anti-CD3/CD28, irrespective of the inflammation scenario (for TNF-α: soluble anti-CD3/CD28 RAT-T vs. PTA-B p = 0.02, soluble anti-CD3/CD28 RAT-T vs. NO INFL-B p = 0.035, immobilized anti-CD3/CD28 RAT-T vs. PTA-B p = 0.02, immobilized anti-CD3/CD28 NO-INFL T vs. PTA-B p = 0.049).

Moreover, we noticed that the pro-inflammatory cytokine TNF-α was highest in PTA-B (for TNF-α: RAT-T vs. PTA-B p = 0.008, PTA-T vs. PTA-B p = 0.03, NO INFL-T vs. PTA-B p = 0.01, Fig. 1 and Table 2). Similarly, IL-4 production was highest in blood samples versus tonsil tissue (RAT-T vs. PTA-B p = 0.0001, PTA-T vs. PTA-B p = 0.0001, NO-INFL-T vs. PTA-B p = 0.0001, Table 2). Notably, IL-6 production was significantly higher in T cells from RAT in comparison to not inflamed tonsil tissue (RAT-T vs. NO INFL-T p = 0.02). However, IL-6 levels generated in tonsillar T cells were low compared to the levels produced by peripheral T cells, suggesting that IL-6 may not be a physiologically relevant cytokine in tonsils (soluble anti-CD3/CD28: RAT-T vs. PTA-B p = 0.008, RAT-T vs. NO INFL-B p = 0.01, PTA-T vs. PTA-B p = 0.03, NO INFL-T vs. PTA-B p = 0.007, NO INFL-T vs. NO INFL-B p = 0.02, immobilized anti-CD3/CD28: RAT-T vs. PTA-B p = 0.03).

In support of a different response potency of tonsillar *versus* peripheral T cells, we noted significant differences in PTA samples with regard to IL-4 production in response to soluble CD3/CD28 (PTA-T vs. PTA-B p = 0.0001), IL-6 release in response to soluble CD3/CD28 (PTA-T vs. PTA-B p = 0.03) and TNF-α release following stimulation with PMA/Ionomycin (PTA-T vs. PTA-B 0.03). Finally, the direct comparison of NO INFL-T and NO INFL-B also revealed significant differences for IL-6 production between tonsillar and peripheral T cells challenged with soluble CD3/CD28 (NO INFL-T vs. NO INFL-B p = 0.02).

## Discussion

The current study documented cytokine release after stimulation of tonsillar T cells from two inflammatory scenarios of tonsillar pathology, i.e. RAT and PTA. The results underscore the notion that the palatine tonsils are a functional immune organ even in adults and adults with chronic inflammatory tonsillar disease.

The strength of this study was the analysis of T cells originating from different forms of inflammation in tonsil side-by-side to non-inflamed tonsil specimens and peripheral lymphocytes from the same patients (the latter limited to a subgroup of donors). At the same time, the small group numbers, inherent to this type of study, was certainly a limitation preventing more robust statistics.

We also took care to compare distinct modes of T cell stimulation, as this process is finely tuned in lymphocytes to discriminate productive antigen-challenge from non-productive cases. Thus, antibody-based clustering of the TCR via CD3 and its co-receptor CD28 is known to trigger productive and full-blown T cell activation (culminating in IL-2 secretion and clonal expansion) only when antibodies are administered immobilized on surfaces^16^. Thus, our stimulation protocol included low-grade TCR stimulation (soluble anti-CD3/CD28) and high-grade stimulation conditions (Abs immobilized on beads), plus a positive control with PMA/ionomycin.

In a previous study, we observed a trend to a higher percentage of T helper cells in the blood of patients with PTA in comparison to RAT. Furthermore, tonsil tissue of RAT contained more PD1(+) CD4(+) T cells reflecting T cell exhaustion due to chronic infection^13^.

TCR stimulation increased the pro-inflammatory cytokines TNF-α, IFN-γ, and IL-2 much higher than of the anti-inflammatory cytokines IL-4 and IL-10 in tonsil and blood, suggesting that a Th1-response predominated over Th2-response. Komorowska et al. reported similar results. In their work, which analyzed cytokine production by lymphoid cells from adenoids and tonsils, the Th1 response dominated over the humoral immune Th2 reaction in palatine tonsils as compared to adenoids in 25 children between 4 and 15 years^17^. In an RNA-PCR-analysis of adults, it was also shown that the Th1 based immune response predominated in patients with chronic inflammatory tonsillitis, whereas the Th2 immune reactions were more pronounced in adenoids of adenoid hyperplasia^19^. In our own present work, Th1 immune response dominated in RAT clearly over Th2 immune response.

Along these lines, pro-inflammatory cytokines dominate in children with chronic tonsillitis not just in lymphoid tissue, but also in saliva^18^. Specifically, levels of TNF-α, IL-1, IL-6 are higher in the saliva of children with chronic tonsillitis, although statistically significant differences were only noted for the pro-inflammatory cytokine IL-6^18^. In our study, IL-6 was also significantly higher in tonsil tissue of RAT than tonsil tissue of PTA and non-inflamed tonsil tissue. However, it was striking that IL-6 production by tonsillar T cells of all scenarios was negligibly low compared to the levels produced by peripheral T cells. Importantly, this feature was specific for IL-6, as other cytokines where secreted to comparable orders of magnitude in lymphocytes from tonsils and blood. We conclude that tonsillar T cells feature a skewed TCR response with low IL-6 production, indicating that IL-6 may not be a particularly relevant cytokine in the context of the tonsillar immune response.

Our findings also document robust secretion of the anti-inflammatory cytokines IL-10 and IL-17 by tonsillar T cells, in accordance with previous reports by others^20,21^. This anti-inflammatory response may be owed to tonsillar follicular T helper cells (Tfh), as Tfh cells are abundant in human tonsils and prolific in the production of IL10^21^. We did not use surface markers to discriminate Thf cells within our T cell preparations, but the findings reported warrant analyzing the contribution of Tfh cells to the tonsillar immune response to infections in future studies.

The present findings are consistent with the concept that human tonsils are immunologically functional entities. Immunomodulatory therapies aiming at the manipulation of the Th1/Th2 balance response are thus a plausible and conceivable new therapeutic strategy in tonsillar inflammation^22^. Immunomodulators with inhibition of Th1 response could contribute to an improvement of RAT. Drugs for inhibition of IL-2 and TNF-α are used for autoimmune diseases like rheumatoid arthritis, Crohn’s disease, colitis ulcerosa, or psoriasis^23^. Such therapies are currently not in use for RAT. This may be owed to the fact that tonsillotomy or tonsillectomy is expected to have fewer systemic side effects compared to immune modulation. However, in the case of contraindications to surgery or in the case of simultaneous autoimmune diseases, immunomodulators could conceivably contribute to an improvement of tonsil inflammatory diseases.

In summary, we suggest that in future immunomodulatory therapies or tonsillotomy, i.e. only a partial resection of the tonsils should be discussed and considered more seriously as an alternative to tonsillectomy in the case of RAT, as tonsillotomy should preserve, at least partially, tonsil immunity. In the case of PTA, abscess incision or cranial tonsillotomy^24^ as an alternative to abscess tonsillectomy could similarly be a promising strategy from an immunological perspective.

## Conclusion

T-cells in palatine tonsils from adult patients with recurrent acute tonsillitis and peritonsillar abscess are functional and responsive and feature a characteristic, specific cytokine response with low IL-6 and features of Th1. We propose that tonsillectomy should be performed only in severe disease with high impairments. Tonsillotomy could be an alternative therapeutic option for preserving tonsillar immunity. Immune-based therapies aiming at the modulation of the Th1/Th2 balance represent a promising new therapeutic concept for exceptional cases of tonsillar inflammation.

## Supplementary information

Supplementary Information.
