# Supplementary material for: Cytokine production in patients with recurrent acute tonsillitis: analysis of tonsil samples and blood
Source: Sci Rep. 2020 Aug 3;10:13006. doi: 10.1038/s41598-020-69981-1 (PMC7400737; doi:10.1038/s41598-020-69981-1)
Supplement: Supplementary file 1 — Supplementary Information. [file 41598_2020_69981_MOESM1_ESM.docx]

**Cytokine production in patients with recurrent acute tonsillitis: analysis of tonsil samples and blood**

Katharina Geißler^1,2*^, Cynthia Weigel^2,†^, Katja Schubert^2,3^, Ignacio Rubio^2,4^, Orlando Guntinas-Lichius^1^

^1^Department of Otorhinolaryngology, Jena University Hospital, Germany

^2^Integrated Research and Treatment Center, Center for Sepsis Control and Care, Jena University Hospital, Germany

^3^Leibniz Institute for Natural Product Research and Infection Biology – Hans Knöll Institute (HKI) Jena, Germany

^4^Department of Anesthesiology and Intensive Care Medicine, Jena University Hospital, Jena, Germany

^†^Current address: Department of Biochemistry and Molecular Biology, Virginia Commonwealth University School of Medicine, Richmond, USA

**Supplementary Figure 1: Cytokine release (pg/ml) from tonsillar (-T) or peripheral (-B) T cells. Purified T cells were either left untreated or stimulated with soluble anti-CD3/CD28 Abs or PMA/ionomycin. Data are presented as box plot; circles stand for patients.**

**

**
